# Efficacy and safety of biologics in erythrodermic psoriasis: a systematic review and single-arm meta-analysis

**DOI:** 10.3389/fimmu.2025.1714587

**Published:** 2025-10-29

**Authors:** Lingjie Gao, Lu Shen, Hongwei Yan, Xinyang Liu, Yiran Wang, Xiaobo Li

**Affiliations:** 1 Department of Dermatology, The First Affiliated Hospital of China Medical University, Shenyang, China; 2 School of Nursing, China Medical University, Shenyang, China; 3 Shengjing Hospital Affiliated to China Medical University, Shenyang, China; 4 Nursing Department, The First Affiliated Hospital of China Medical University, Shenyang, China

**Keywords:** erythrodermic psoriasis, biologics, efficacy, safety, PASI 75

## Abstract

**Introduction:**

Erythrodermic psoriasis (EP) is a rare but severe inflammatory skin disease that affects the whole body. It presents clinically as widespread redness, scaling, and systemic symptoms. Treatment choices are currently limited, and conventional drugs often raise safety issues, highlighting the need for more effective and safer treatments. Biologics have emerged as a new approach. This study aimed to systematically evaluate the efficacy and safety of different biologics for EP by conducting a systematic review and single-arm meta-analysis.

**Methods:**

We performed a systematic search of four databases—Embase, PubMed, Scopus, and the Cochrane Library—for relevant clinical studies published up to May 2025. Treatment effects were evaluated by analyzing statistical data such as single-arm PASI 75 response rates and their 95% confidence intervals (CI). Heterogeneity was assessed using the I² statistic, and subgroup analyses were conducted based on drug targets and treatment duration. A random-effects meta-analysis was applied to quantitatively synthesize the data. All statistical analyses were performed using Stata 18.0 software.

**Results:**

A total of 18 studies involving 342 patients were included in the analysis. Patients receiving IL-17-targeted biologics achieved higher PASI 75 response rates compared to those treated with TNF-α or IL-23-targeted biologics. Response rates for IL-17-targeted agents continued to climb over 12 weeks, peaking at 82% by week 16, indicating superior efficacy in improving skin lesions compared to other biologic categories. IL-23-targeted biologics exhibited the lowest incidence of adverse events (5%, 95% confidence interval: 4%-13%), suggesting superior safety compared to IL-17 and TNF-α-targeted therapies.

**Discussion:**

This single-arm meta-analysis demonstrates the efficacy and safety of biologics in treating erythrodermic psoriasis (EP). These treatments are particularly valuable for rapid control and long-term management of the disease. Further large-scale studies with long-term follow-up are needed to confirm their benefits in specific patient groups and for preventing recurrence.

## 1 Introduction

Erythrodermic psoriasis (EP) is a rare and severe inflammatory skin disease (1). Clinical manifestations include generalized diffuse erythema affecting more than 75% of the body surface area(BSA) (2), accompanied by extensive desquamation involving nearly all exposed skin (3). Systemic symptoms like fever, lymphadenopathy, and electrolyte imbalances are common (4). In severe cases, the condition can progress to multiple organ failure, substantially increasing the risk of mortality (5). Epidemiological studies show significant geographical and demographic differences in EP prevalence, with higher rates observed in high-income regions and among elderly populations. This pattern highlights the complexity of managing the disease and the need for effective treatments.

The development of erythrodermic psoriasis can be initiated by various triggers, such as sudden withdrawal or rapid tapering of corticosteroids and other systemic therapies, drug hypersensitivity reactions, systemic infections, and psychological stress (6). Pathophysiologically, erythrodermic psoriasis is characterized by a more severe and systemic inflammatory response compared to plaque psoriasis, featuring marked vascular alterations and elevated intercellular adhesion molecule expression in affected skin (7). Central to this process is the aberrant activation of dendritic cells, which drives overactivation of the interleukin-23 (IL-23)–Th17 axis and subsequent release of effector cytokines, including tumor necrosis factor-α (TNF-α) (8). These cytokines create a complex inflammatory network that further amplifies immune responses, promoting dysregulated keratinocyte proliferation and impaired differentiation. Ultimately, this leads to widespread erythema, scaling, and compromised skin barrier function across the body, often accompanied by severe systemic complications (9).

Erythrodermic psoriasis (EP) is currently managed with a range of treatments, including topical agents, phototherapy, conventional systemic drugs, and biologics. Topical medications are appropriate for mild cases but have limited effect in moderate-to-severe disease. Conventional systemic therapies, such as methotrexate and cyclosporine, can improve lesions in moderate-to-severe patients; however, long-term use carries risks of serious adverse events, including liver and kidney toxicity—which often restrict their clinical utility and negatively impact treatment adherence and patient satisfaction (10). These limitations highlight the ongoing challenges in EP management and emphasize the urgent need for novel therapies that are both more effective and safer.

In recent years, biologics have shown important progress in the treatment of psoriasis and other autoimmune diseases. By precisely targeting and regulating abnormal immune pathways, these drugs not only improve treatment outcomes but also help overcome the safety limitations of conventional therapies. Biologics have shown favorable results across different subtypes of psoriasis. The Psoriasis Area and Severity Index (PASI) is the main measure of treatment success, and PASI 75 response—defined as at least a 75% reduction from the baseline PASI score—is widely used as a key efficacy endpoint in clinical trials (11, 12).Nevertheless, there is still a lack of comprehensive evidence from systematic reviews and single-arm meta-analyses focusing specifically on the use of biologics in the erythrodermic psoriasis (EP) subtype.

Therefore, this study systematically evaluates the efficacy and safety of various biologics—specifically IL-17, TNF-α, and IL-23 inhibitors—in patients with erythrodermic psoriasis (EP) by integrating available clinical evidence. The main goal is not only to confirm the overall role of biologics in EP treatment, but also to directly compare differences in efficacy and safety among these drug classes at key time points. The goal was to offer reliable, evidence-based guidance for clinical decision-making, with the ultimate aim of improving treatment outcomes and patient quality of life.

## 2 Method

### 2.1 Literature search

On May 26, 2025, the authors searched four electronic databases—PubMed, Scopus, Embase, and the Cochrane Library—using a combination of keywords related to erythrodermic psoriasis, biologics, and clinical trials. PubMed was used as an example, and retrieval formula is provided in **Supplementary Table 1**. A manual search of relevant sources was also performed. The study followed the Preferred Reporting Items for Systematic Reviews and Meta-Analyses (PRISMA) guidelines (13), and was registered in PROSPERO (CRD420251129658).

### 2.2 Inclusion and exclusion criteria

Inclusion Criteria: (1) Patients clinically diagnosed with erythrodermic psoriasis; (2) Patients receiving initial therapy with biologics or who have previously received biologic therapy; (3) Reporting of efficacy evaluation indicators such as the Psoriasis Area and Severity Index (PASI) or adverse events for erythrodermic psoriasis. Exclusion Criteria: (1) Literature classified as reviews or commentaries; (2) Duplicate publications or updated literature; (3) Literature where full text was unavailable or data were incomplete.

### 2.3 Efficacy evaluation indicators

Clinical efficacy was assessed using the Psoriasis Area and Severity Index (PASI) 75 response as the primary outcome measure, reported as the proportion of patients who achieved a PASI 75 improvement.

### 2.4 Data extraction and risk of bias assessment

The following characteristics were extracted from the included studies: author, region, ethnicity, sample size, age, sex, treatment regimen, and outcome measures. Study quality was evaluated using the ROBINS-I tool (14) and the JBI Critical Appraisal Tool for Case Series (15). Any disagreements between researchers were resolved through discussion. Unresolved issues were referred to a relevant expert, with third-party arbitration used if consensus could not be reached.

### 2.5 Statistical analysis

Treatment efficacy was analyzed using single-arm proportion rates with 95% confidence intervals (CI). Heterogeneity was evaluated with the I² statistic, and subgroup analyses were performed by drug target and treatment duration. A fixed-effect model was used when I² ≤ 50% and p ≥ 0.1; otherwise, a random-effects model was applied. All tests were two-sided, with statistical significance set at p < 0.05. Publication bias was assessed using Egger’s test, where p > 0.05 suggested no significant bias. Sensitivity analysis was conducted to examine the robustness of the pooled results. All analyses were carried out using Stata 18.0 software.

## 3 Result

### 3.1 Study characteristics

**Figure 1:**
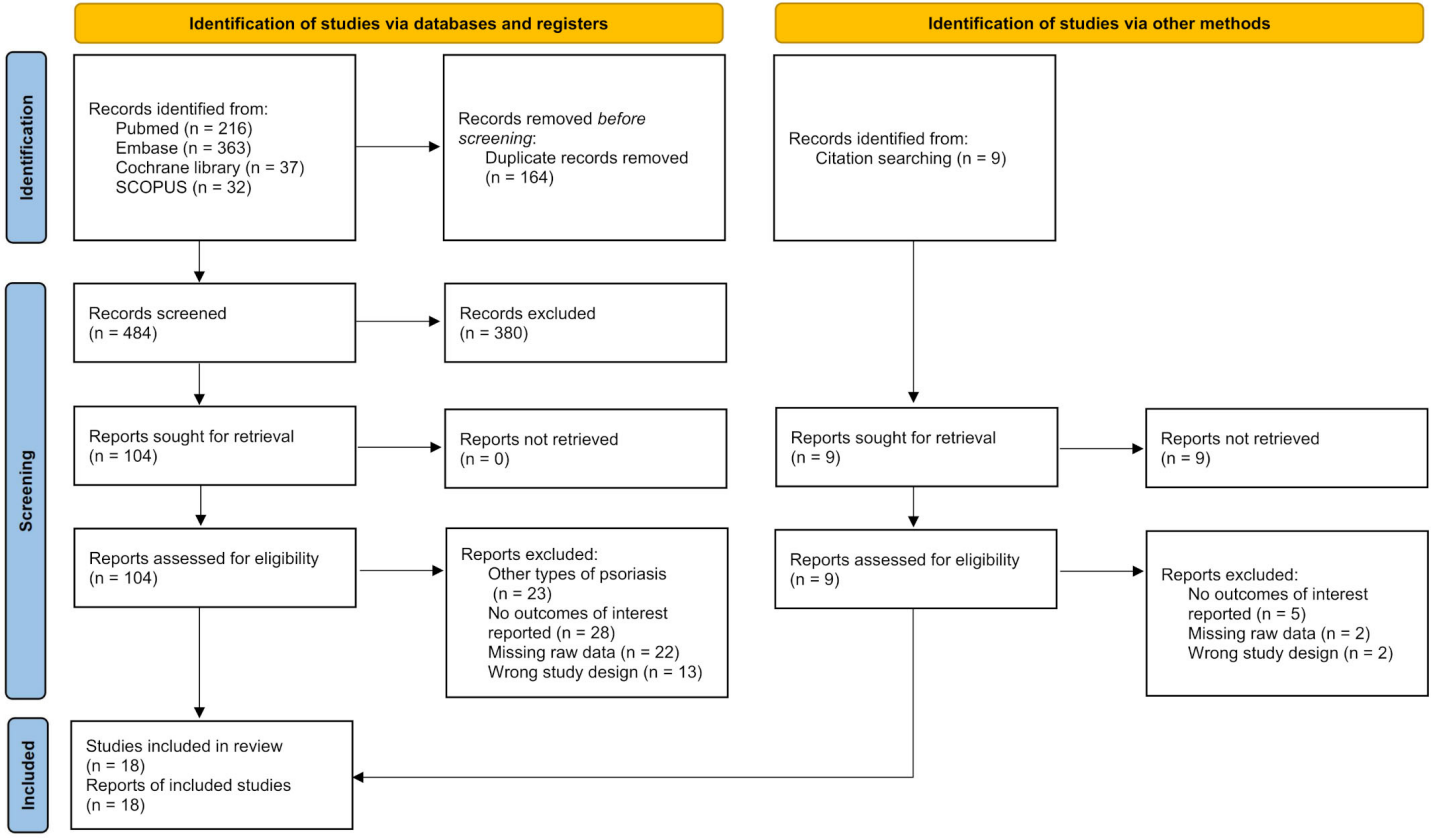
Study flow diagram.

Of the 648 studies identified, 18 met the inclusion criteria (16–33) (**Figure 1**), involving a total of 342 patients. Among them, 194 were treated with IL-17-targeted biologics (secukinumab, ixekizumab,Brodalumab), 63 received TNF-α-targeted biologics (etanercept, infliximab, adalimumab, certolizumab), and 63 were administered IL-23-targeted biologics (guselkumab, ustekinumab, risankizumab). Baseline data and key characteristics of the included studies are summarized in **Table 1**. (see **Table 1** for details).

**Table 1:** Characteristics of included studies.

Author	Number of Participants	Male	Region	BMI	Age	Average disease duration, years	Study type	Drug	Targeted therapy	Outcome measure	Duration	Baseline PASI
Bhatnagar 2023 (16)	9	7	India		56(14.6)^1^	15.4(10.2)^1^	Prospective Study	Secukinumab	IL-17	PASI, Body Surface Area(BSA), AE	24W	44.9(9.4)^1^
Chiang 2021 (17)	13	12	China	24.6(4.1)^1^	50.5(15.3)^1^	24.2 (13.3)^1^	Case Series	Guselkumab	IL-23	PASI, Drug Survival	28W	23.8 (8.7)^1^
Damiani 2019 (18)	13	9	International	4 (22-27)2	40 (28-52)2		Retrospective Study	Secukinumab	IL-17	PASI, Dermatology Life Quality Index(DLQI), AE	52W	10 (7–15)3
Esposito 2006 (19)	10	8	Italy		56.4(11.1)^1^	1.3(1.3)^1^	Prospective Study	Etanercept	TNF-α	PASI, AE	24W	39.8(15.2)^1^
Hsu 2025 (20)	22	16	Taiwan	28.5(4.6)^1^	49.7(12.8)^1^	23.7 (10.7)^1^	Retrospective Study	Risankizumab	IL-23	PASI, Drug Survival, AE	100W	20.7 (7.8)^1^
Lo 2021 (21)	14	10	China		48.6(13.2)^1^	20.2 (10.4)^1^	Retrospective Study	Ixekizumab	IL-17	PASI, BSA, AE	52W	32.3 (12.6)^1^
Morita 2022 (22)	5	3	Japan	28.1(2.8)^1^	42.2(14.4)^1^	0.4^4^	Prospective Study	Ixekizumab	IL-17	Global improvement score(GIS), Physician Global Assessment(PGA), Psoriasis Severity Index(PSSI), PASI, BSA, DLQI, AE	20W	41.1^4^
Okubo 2022 (23)	15	13	Japan	400mg:25.6(4.1)^1^ 200mg:26.3(5.1)^1^	400mg:47.8(11.3)^1^ 200mg:56.0(8.5)^1^	400mg:4.7 (5.3) ^1^ 200mg:9.0 (10.7)^1^	Prospective Study	Certolizumab	TNF-α	PGA, Clinical Global Impressions scale- Improvement(CGI-I), PASI, DLQI, AE	52W	400mg:43.7(17.9)^1^ 200mg:34.3 (9.2)^1^
Panda 2021 (24)	6	4	India		37.3(6.9)^1^	6.2(2.2)^1^	Case Series	Secukinumab	IL-17	PASI, DLQI, Visual Analogue Scale(VAS)	52W	26.0(4.0)^1^
Plana 2024 (25)	28	22	Spain	27.8 (5.7)^1^	52.6 (16.8)^1^	22.75 (13.6)^1^	Retrospective Study	Ustekinumab	IL-23	PASI, BSA, AE	148W	43.1 (12.3)^1^
Reymundo 2023 (26)	16	11	Portugal Spain	29.4 (4.8)^1^	54.81 (17.1)^1^	17.9 (16.4)^1^	Retrospective Study	Secukinumab	IL-17	PASI, DLQI, PGA, AE	144W	35.5(2.7)^1^
Saeki 2017 (27)	8	7	Japan		50.2(12.9)^1^	18.4(14.0)^1^	Prospective Study	Ixekizumab	IL-17	PASI, DLQI, PGA, BSA, GIS, PSSI, Itch Numeric Rating Scale(INRS), AE	52W	42.8(11.6)^1^
Viguier 2012 (28)	28	20	France	25.2 (18.4-49)^2^	40 (19-73)^2^		Retrospective Study	Etanercept Infliximab	TNF-α TNF-α	PASI, BSA, DLQI, PGA, AE	96W	38(20-65)^2^
Wang 2025 (29)	20	12	China	26.2(7.0)^1^	43.8(16.9)^1^	13.7(11.7)^1^	Retrospective Study	Adalimumabs Secukinumab	TNF-α IL-17	PASI, AE	24W	45.4(8.8)^1^
Weng 2018 (30)	10	8	China		42.6(11.0)^1^	20.6(11.4)^1^	Case Series	Secukinumab	IL-17	PASI, BSA	24W	32.4(5.7)^1^
Yamasaki 2017 (31)	18	14	Japan	24.3(4.9)^1^	50.8(12.2)^1^	6.37(10.1)^1^	Prospective Study	Brodalumab	IL-17	CGI-I, PASI, DLQI, PGA, BSA, AE	52W	36.1(13.1)^1^
Zhang 2022 (32)	7	7	China		51.4(15.8)^1^	3.8(3.8)^1^	Case Series	Secukinumab	IL-17	PASI, BSA, AE	90W	42.2(12.1)^1^
Zhou 2024 (33)	160	128	China	24.2 (22.2,26.0)^3^	48.2(16.5)	10.0 (0, 19.0)^3^	Retrospective Study	Secukinumab	IL-17	PASI, BSA, AE	24W	24.7 (15.2,38.7)^3^

1 Reported as mean (SD).

2 Reported as median (range).

3 Reported as median (IQR).

4 Reported as mean.

### 3.2 Risk of bias assessment

The ROBINS-I tool is designed for use with non-randomized studies that evaluate intervention effects. It is mainly used to assess the methodological quality of case reports and case series, and is commonly applied in systematic reviews to evaluate risk of bias in observational studies (see **Table 2**, **Table 3**).

**Table 2:** Risk of bias assessment for non-randomized controlled studies.

Research	Type of bias							Overall risk
	①	②	③	④	⑤	⑥	⑦	
Bhatnagar 2023 (16)	Moderate	Moderate	Low	Low	Low	Low	Low	Moderate
Damiani 2019 (18)	Moderate	Moderate	Low	Low	Moderate	Low	Low	Moderate
Esposito 2006 (19)	Severe	Moderate	Low	Low	Low	Moderate	Low	High
Hsu 2025 (20)	Moderate	Moderate	Low	Low	Moderate	Low	Low	Moderate
Lo 2021 (21)	High	Moderate	Low	Moderate	Moderate	Low	Low	High
Morita 2022 (22)	Low	Moderate	Low	Low	Low	Moderate	Low	Moderate
Okubo 2022 (23)	High	High	Low	Low	Low	Moderate	Low	High
Plana 2024 (25)	Moderate	Moderate	Low	Moderate	Low	Low	Moderate	Moderate
Reymundo 2023 (26)	Moderate	Low	Low	Moderate	Moderate	Low	Low	Moderate
Saeki 2017 (27)	Low	Low	Low	Low	Low	Moderate	Low	Moderate
Viguier 2012 (28)	Severe	Moderate	Low	Moderate	Severe	Low	Low	High
Yamasaki 2017 (31)	Moderate	Low	Low	Moderate	Low	Moderate	Low	Moderate
Wang 2025 (29)	Moderate	Moderate	Low	Low	Low	Low	Low	Moderate
Zhou 2024 (33)	Moderate	Low	Low	Low	Moderate	Moderate	Low	Moderate

①Bias due to Confounding.

②Bias in Selection of Participants into the Study.

③Bias in Classification of Interventions.

④Bias due to Deviations from Intended Interventions.

⑤Bias due to Missing Data.

⑥Bias in Measurement of Outcomes.

⑦Bias in Selection of the Reported Result.

**Table 3:** Risk assessment of bias in case series studies.

Research	Type of bias										Overall risk
	①	②	③	④	⑤	⑥	⑦	⑧	⑨	⑩	
Chiang2021 (17)	Yes	Yes	Yes	Yes	Yes	Yes	es	Yes	Yes	Yes	Low
Panda2021 (24)	Yes	Yes	Yes	No	No	Yes	Yes	Yes	No	Yes	Low
Weng2018 (30)	Yes	Yes	Yes	No	No	Yes	Yes	Yes	No	Yes	Moderate
Zhang2022 (32)	Yes	Yes	Yes	No	No	Yes	Yes	Yes	No	Yes	Moderate

①Were there clear criteria for inclusion in the case series?

②Was the condition measured in a standard, reliable way for all participants included in the case series?

③Were valid methods used for identification of the condition for all participants included in the case series?

④Did the case series have consecutive inclusion of participants?

⑤Did the case series have complete inclusion of participants?

⑥Was there clear reporting of the demographics of the participants in the study?

⑦Was there clear reporting of clinical information of the participants?

⑧Were the outcomes or follow-up results of cases clearly reported?

⑨Was there clear reporting of the presenting sites’/clinics’ demographic information?

⑩Was statistical analysis appropriate?

### 3.3 PASI

#### 3.3.1 PASI 75 response rate at 12 weeks

**Figure 2:**
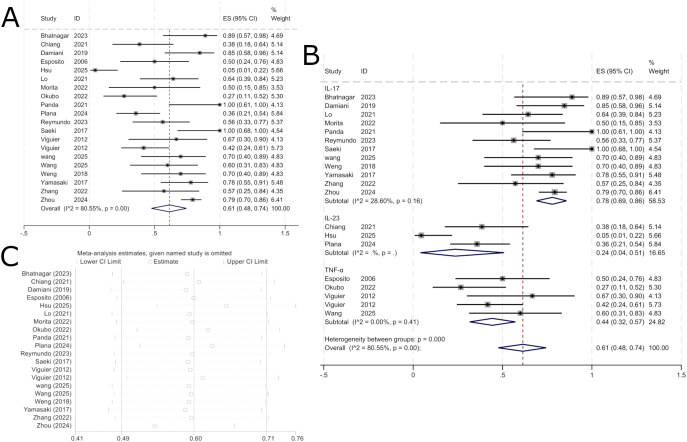
Pooled analysis of response rate for PASI 75 within 12 weeks. **(A)** Forest plot of PASI 75 response rate within 12 weeks. **(B)** Forest Plot of PASI 75 Response Rate Subgroup Analysis Within 12 Weeks. **(C)** Forest Plot of PASI 75 Response Rate Subgroup Analysis Within 12 Weeks.

A total of 18 publications, comprising 20 study groups, provided outcome data at 12 weeks for 342 patients, among whom 210 achieved a PASI 75 response. The pooled analysis showed that the overall PASI 75 response rate for biologic therapy in erythrodermic psoriasis at 12 weeks was 61% (95% CI: 40%–73%; **Figure 2A**). The specific drugs and number of studies in each subgroup are as follows: IL-17 inhibitors include secukinumab (n=8), ixekizumab (n=3), and brodalumab (n=1); IL-23 inhibitors include ustekinumab (n=1), risankizumab (n=1), and guselkumab (n=1); TNF-α inhibitors included etanercept (n=2), certolizumab (n=1), adalimumab (n=1), and infliximab (n=1).

In subgroup analysis by biologic target, the PASI 75 response rate was 78% (95% CI: 69%–86%) for IL-17-targeted biologics, 44% (95% CI: 32%–57%) for TNF-α-targeted biologics, and 24% (95% CI: 4%–51%) for IL-23-targeted biologics. As shown in **Figure 2B**, IL-17-targeted biologics were the most effective in achieving PASI 75 improvement over 12 weeks, followed by TNF-α-targeted biologics, while IL-23-targeted biologics showed the lowest response. Due to substantial heterogeneity (I² = 80.55%), a random-effects model was used. Sensitivity analysis indicated that the results were robust and stable across studies (**Figure 2C**).

#### 3.3.2 PASI 75 response rate at 16 weeks

**Figure 3:**
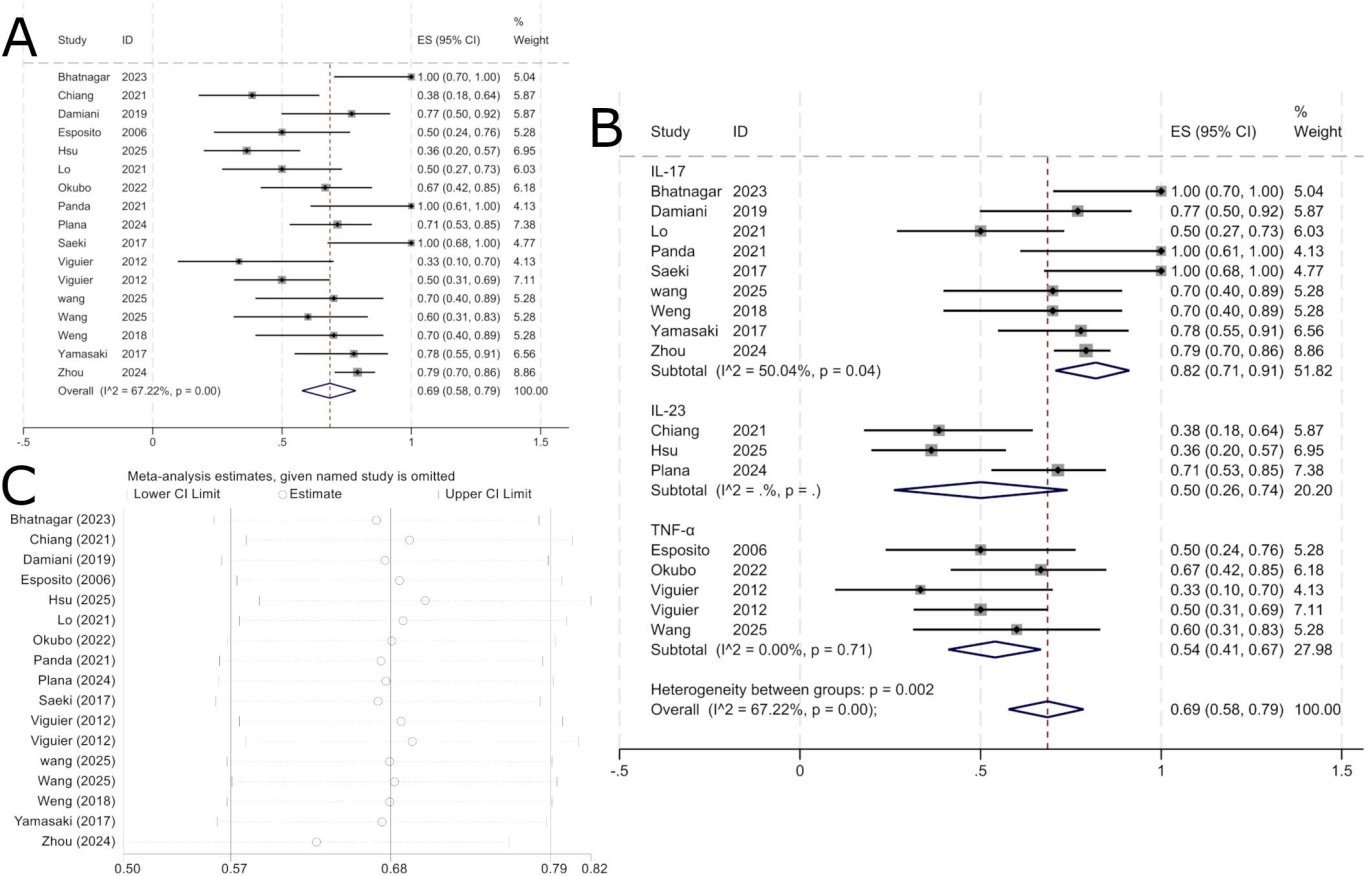
Pooled analysis of response rate for PASI 75 within 16 weeks. **(A)** Forest plot of PASI 75 response rate within 16 weeks. **(B)** Forest Plot of PASI 75 Response Rate Subgroup Analysis Within 16 Weeks. **(C)** Forest Plot of PASI 75 Response Rate Subgroup Analysis Within 16 Weeks.

A total of 15 publications encompassing 17 study groups provided comparative outcome data at 16 weeks, involving 315 patients, of whom 216 achieved a PASI 75 response rate. Pooled analysis indicated that the PASI 75 response rate for biologic therapy in erythrodermic psoriasis within 16 weeks was 69% (95% CI: 40%-73%), as shown in **Figure 3A**.Subgroup analyses were conducted for different targeted biologics used, with specific drugs and study numbers in each subgroup as follows: IL-17 inhibitors included secukinumab (n=6), ixekizumab (n=2), and brodalumab (n=1); IL-23 inhibitors included ustekinumab (n=1), risankizumab (n=1), and guselkumab (n=1); TNF-α inhibitors included etanercept (n=2), certolizumab (n=1), adalimumab (n=1), and infliximab (n=1).

Results showed that the PASI 75 response rate was 82% (95% CI: 71%-91%) among patients receiving IL-17-targeted biologics, while the PASI 75 response rate for IL-23-targeted biologics was 50% (95% CI: 26%-74%), and for TNF-α-targeted biologics it was 54% (95% CI: 41%-67%). As shown in **Figure 3B**, this indicates that over 16 weeks, IL-17-targeted biologics demonstrated the most effective PASI 75 improvement in treating erythrodermic psoriasis, followed by TNF-α-targeted biologics, while IL-23-targeted biologics showed the poorest efficacy. Since I² = 67.22% > 50%, a random-effects model was selected for analysis. Sensitivity analysis was conducted to examine sources of heterogeneity, revealing good stability in the results, as shown in **Figure 3C**.

#### 3.3.3 PASI 75 response rate at 24 weeks

**Figure 4:**
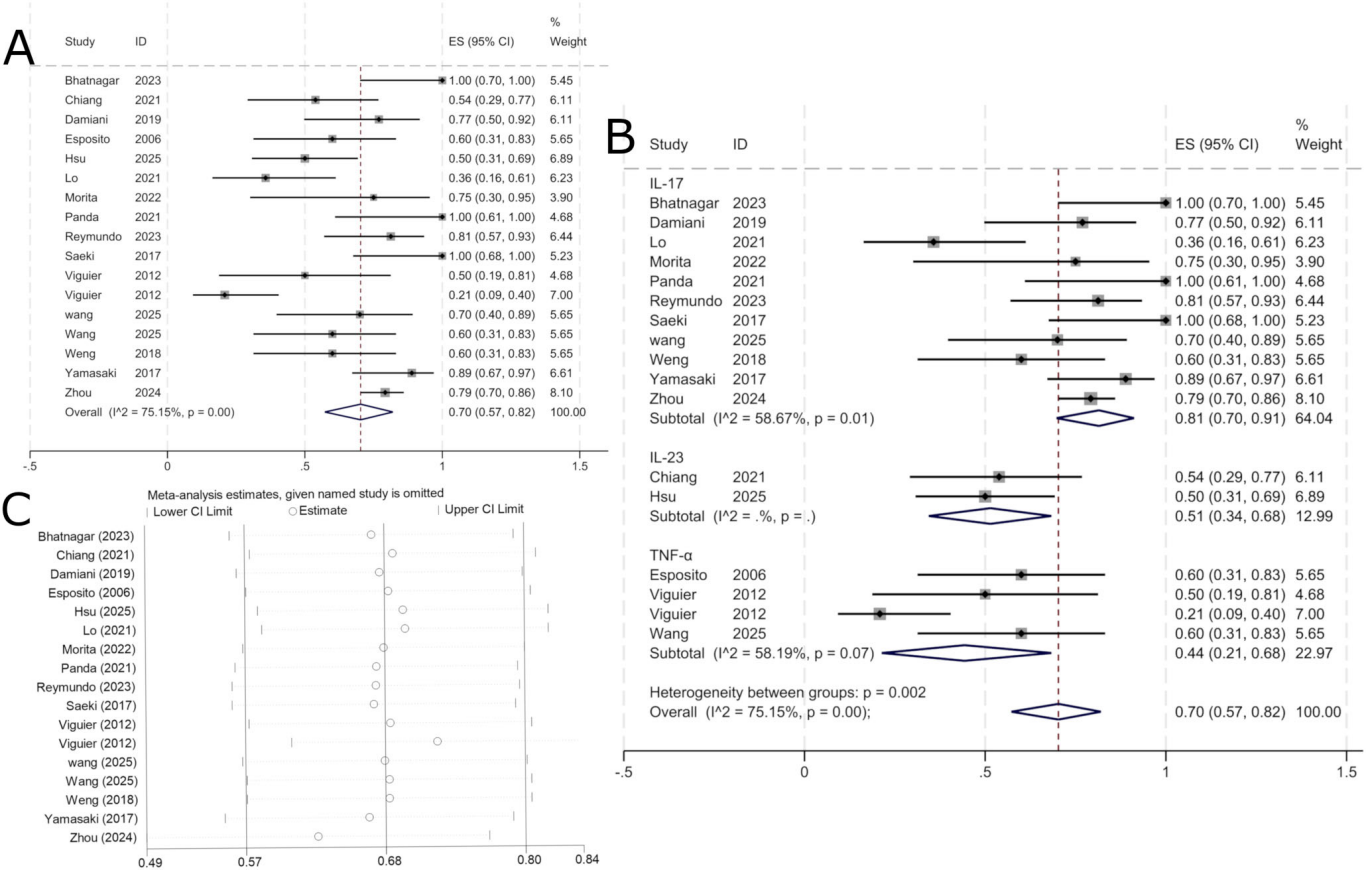
Pooled analysis of response rate for PASI 75 within 24 weeks. **(A)** Forest plot of PASI 75 response rate within 24 weeks. **(B)** Forest Plot of PASI 75 Response Rate Subgroup Analysis Within 24 Weeks. **(C)** Forest Plot of PASI 75 Response Rate Subgroup Analysis Within 24 Weeks.

A total of 15 publications, comprising 17 study groups, reported outcome data at 24 weeks for 291 patients, of whom 203 achieved a PASI 75 response. The pooled analysis indicated a PASI 75 response rate of 70% (95% CI: 57%–82%) for biologic therapy in erythrodermic psoriasis at 24 weeks (**Figure 4A**). The specific drugs and number of studies in each subgroup are as follows: IL-17 inhibitors include secukinumab (n=7), ixekizumab (n=4), and brodalumab (n=1); IL-23 inhibitors include risankizumab (n=1) and guselkumab (n=1); TNF-α inhibitors included etanercept (n=2), adalimumab (n=1), and infliximab (n=1).

Subgroup analysis by biologic target showed PASI 75 response rates of 81% (95% CI: 70%–91%) for IL-17-targeted biologics, 51% (95% CI: 34%–68%) for IL-23-targeted biologics, and 44% (95% CI: 21%–68%) for TNF-α-targeted biologics. As shown in **Figure 4B**, IL-17-targeted biologics demonstrated the highest efficacy over 24 weeks, followed by IL-23-targeted biologics, while TNF-α-targeted biologics showed the lowest response. Given the substantial heterogeneity (I² = 75.15%), a random-effects model was used. Sensitivity analysis confirmed the stability of the results (**Figure 4C**).

#### 3.3.4 PASI 75 response rate after 24 weeks

**Figure 5:**
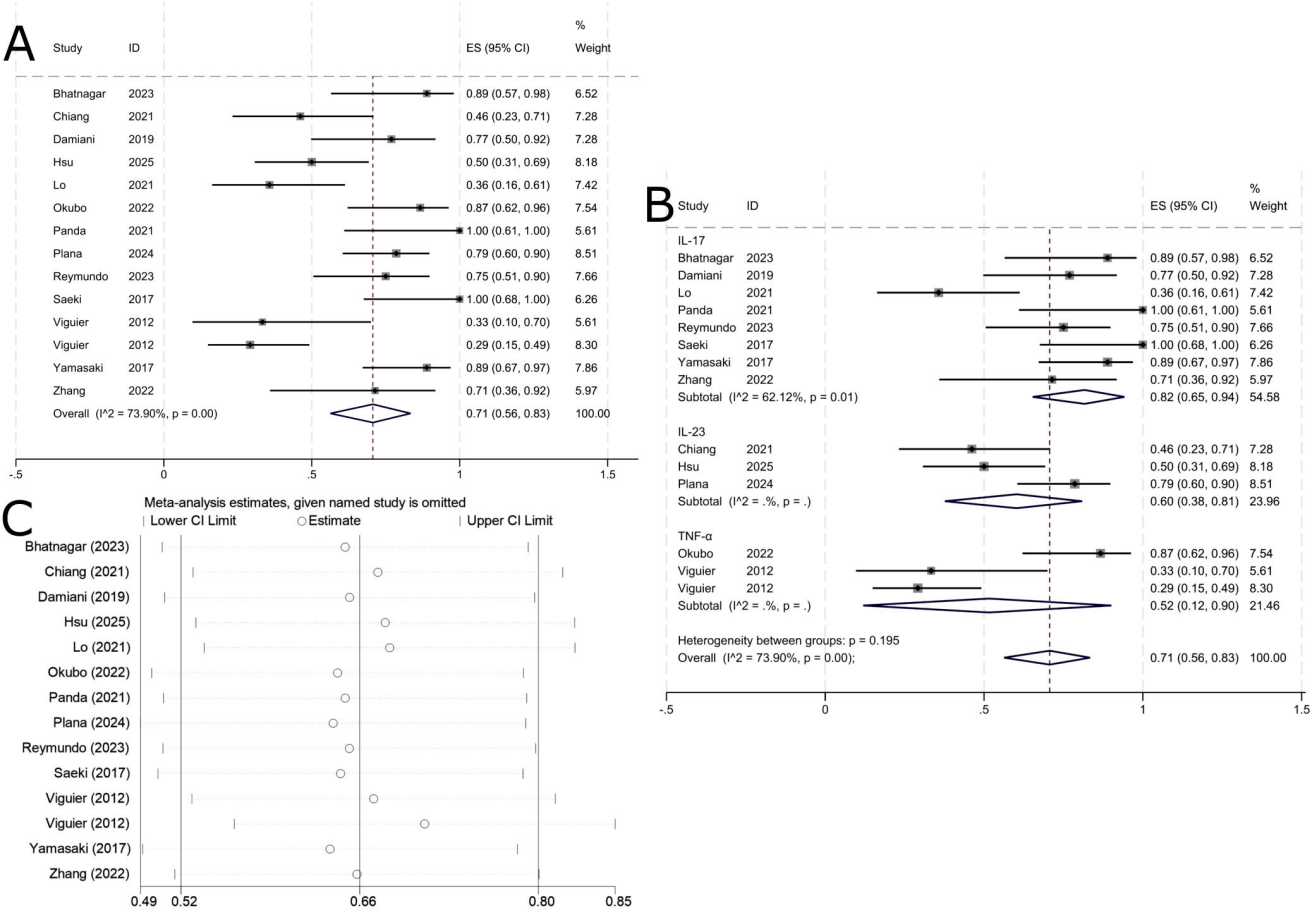
Pooled analysis of response rate for PASI 75 after 24 weeks. **(A)** Forest plot of PASI 75 response rate after 24 weeks. **(B)** Forest Plot of PASI 75 Response Rate Subgroup Analysis after 24 Weeks. **(C)** Forest Plot of PASI 75 Response Rate Subgroup Analysis after 24 Weeks.

This analysis pooled data from studies with extended follow-up (28–148 weeks) to assess the medium- and long-term efficacy of biologics (**Table 1**).The study comprised 13 literature sources, comprising 14 study groups, provided outcome data at 24 weeks for 197 patients, among whom 139 achieved a PASI 75 response. The pooled PASI 75 response rate for biologic therapy in erythrodermic psoriasis at 24 weeks was 71% (95% CI: 56%–83%; **Figure 5A**). The specific drugs and number of studies in each subgroup are as follows: IL-17 inhibitors include secukinumab (n=5), ixekizumab (n=2), and brodalumab (n=1); IL-23 inhibitors include ustekinumab (n=1), risankizumab (n=1), and guselkumab (n=1); TNF-α inhibitors included etanercept (n=1), certolizumab (n=1), and infliximab (n=1).

Subgroup analysis by biologic target showed PASI 75 response rates of 82% (95% CI: 65%–94%) for IL-17-targeted biologics, 60% (95% CI: 38%–81%) for IL-23-targeted biologics, and 52% (95% CI: 12%–90%) for TNF-α-targeted biologics. As illustrated in **Figure 5B**, IL-17-targeted biologics demonstrated the highest efficacy after 24 weeks, followed by IL-23-targeted biologics, while TNF-α-targeted biologics showed the lowest response. Due to substantial heterogeneity (I² = 73.90%), a random-effects model was used. Sensitivity analysis indicated stable results across the included studies (**Figure 5C**).

#### 3.3.5 Adverse events

**Figure 6:**
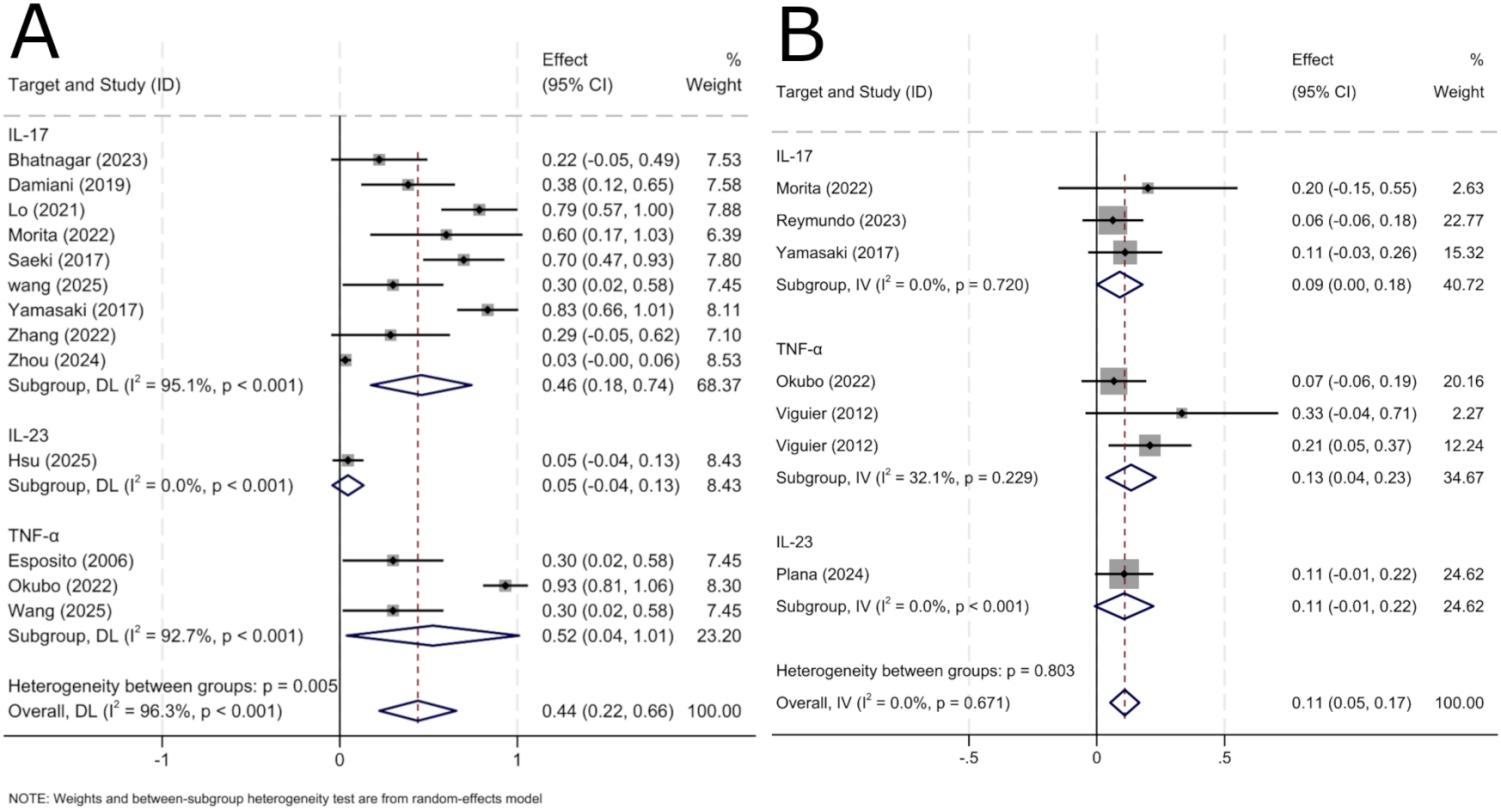
Pooled analysis of adverse event. **(A)** Forest Plot of Overall Adverse Event Incidence by Subgroup Analysis. **(B)** Forest Plot of Subgroup Analysis for Serious Adverse Event Incidence.

The safety analysis included 12 publications (13 study groups) reporting adverse events in erythrodermic psoriasis patients treated with biologics. The overall incidence of adverse events was 44% (95% CI: 22%–66%). Subgroup analysis by drug class showed incidence rates of 46% (95% CI: 18%–74%) for IL-17-targeted biologics, 5% (95% CI: –4%–13%) for IL-23-targeted biologics, and 52% (95% CI: 4%–101%) for TNF-α-targeted biologics (**Figure 6A**). Severe adverse events occurred in 11% (95% CI: 5%–17%) of patients (**Figure 6B**). Commonly reported adverse events included infections, injection site reactions, infusion-related reactions, and diarrhea. Candida infections and injection site reactions were more frequent with IL-7 inhibitors, whereas infusion reactions and serious infections were more commonly associated with TNF-α inhibitors.

### 3.4 Publication bias analysis

Publication bias was assessed using Egger’s test for the included studies. No significant publication bias was observed for PASI 75 at 12 weeks (p = 0.529), 16 weeks (p = 0.410), 24 weeks (p = 0.158), 24 weeks or longer (p = 0.396), or the incidence of serious adverse events (p = 0.050), as all p-values were ≥ 0.05 (**Supplementary Figures 1-5**). In contrast, significant publication bias was detected for the overall adverse event rate (p = 0.017), as shown in **Supplementary Figures 6**.

All the results mentioned above are summarized in **Table 4**.

**Table 4:** Summary of meta-analysis results.

Result	Study	Heterogeneity		ES(95%CI)	P	P(Egger)
		P	I^2^			
Within 12W, PASI 75(%)	18	0.000	80.55	61 (95%CI:40%-73%)	0.000	0.529
Within 16W, PASI 75(%)	15	0.002	67.22	69 (95%CI:40%-73%)	0.000	0.410
Within 24W, PASI 75(%)	15	0.002	75.15	70 (95%CI:57%-82%)	0.000	0.158
Within 24W, PASI 75(%)	13	0.195	73.9	71 (95%CI:56%-83%)	0.000	0.396
TAE(%)	13	0.005	96.3	44 (95%CI:22%-66%)	0.000	0.017
SAE(%)	6	0.803	0.0	0.11 (95%CI:4%-23%)	0.671	0.050

## 4 Discussion

This study evaluated the efficacy and safety of biologics in erythrodermic psoriasis (EP) by means of a systematic review and single-arm meta-analysis. EP is a severe, potentially life-threatening systemic inflammatory skin disease. Its pathogenesis involves abnormal activation of multiple inflammatory pathways and complex cytokine regulation. Current evidence indicates that EP is mainly driven by widespread Th1 and Th17 immune activation, with key roles played by cytokines such as TNF-α, IL-17, and IL-23 in disease initiation and progression (1, 34). Specifically, TNF-α acts as an important early mediator in EP inflammation and further promotes the release of IL-17 and IL-23, creating a positive feedback loop that amplifies systemic inflammation (35). IL-17, a proinflammatory cytokine produced by Th17 cells and type 3 innate lymphoid cells (ILC3), is a central player in EP pathogenesis. It promotes neutrophil activation and infiltration, thereby intensifying the inflammatory response (36, 37). IL-23 is highly expressed in EP lesions and works together with TNF-α to stimulate the proliferation and activation of Th17 cells (38, 39). This leads to increased production of IL-17A and IL-17F, resulting in excessive cell proliferation and abnormal differentiation (40), which together contribute to disease progression.

This study found that biologics targeting different pathways are all effective in treating erythrodermic psoriasis. During the 12-week treatment period, the proportion of patients achieving a PASI 75 response increased gradually over time. Subgroup analysis by target showed that IL-17-targeted biologics performed particularly well in improving skin lesion severity, with the highest response rates observed at 16 and 24 weeks, indicating superior skin clearance. Previous studies have reported complete skin clearance in EP patients following treatment with IL-17-targeted biologics (41), supporting the central role of IL-17 as a key cytokine and aligning with the present findings.

The enrolled patient population demonstrated considerable heterogeneity in baseline characteristics. Patients receiving IL-17 inhibitors tended to present with more severe disease at baseline, which further underscores the notable efficacy observed with this drug class. Variations in prior biologic exposure were also evident across treatment groups, with the IL-23 inhibitor subgroup including a higher proportion of patients with refractory disease, potentially contributing to the lower early response rates observed. Furthermore, differences in demographic factors such as age and ethnicity, as well as comorbidities like metabolic syndrome and prior infections, may have indirectly influenced outcomes by altering drug metabolism or immune response. These baseline imbalances, likely reflecting real-world clinical selection, suggest that the efficacy and safety profiles identified in this analysis are contextual and should be interpreted with these factors in mind. These findings highlight the importance of stratified study designs in future research and individualized treatment selection based on specific patient characteristics.

In terms of treatment trajectory, TNF-α inhibitors showed a better response than IL-23 inhibitors at weeks 12 and 16. With longer treatment duration, however, IL-23 inhibitors gradually overtook TNF-α inhibitors in efficacy by week 24 and beyond. This pattern may be explained by the characteristically slower onset of action of IL-23-targeted therapy. Previous studies have shown that the superior efficacy of anti-IL-23 agents in plaque psoriasis often becomes fully apparent over extended treatment periods, such as 24 to 52 weeks (42). Consistent with this profile, the present analysis also indicated a clear increase in response rates for IL-23 inhibitors at and beyond week 24.

This study also noted a decrease or fluctuation in the PASI 75 response rate for anti-TNF-α agents by week 24 and beyond compared to earlier time points. Several factors may explain this observation. First, the systemic hyperinflammation seen in erythrodermic psoriasis may promote the development of anti-drug antibodies, which can reduce drug activity or accelerate clearance, thereby impairing treatment efficacy (43). Second, in some cases, TNF-α inhibitors may trigger or worsen other immune-mediated inflammatory conditions, such as new or aggravated psoriasiform skin lesions or arthritis (44). Such events could be recorded as reduced treatment response in clinical evaluations. In addition, the analyzed population may have included patients with refractory disease who did not respond adequately to prior conventional or biologic therapies, and who may thus show limited response to TNF-α inhibition. Therefore, the apparent decline in late-stage efficacy of anti-TNF-α agents likely reflects the complexity of treating this severe and often refractory EP population, rather than an inherent lack of efficacy.

Erythrodermic psoriasis (EP) presents clinically as widespread redness, swelling, and scaling that can affect the entire or nearly entire skin surface. It is often accompanied by severe itching, pain, and systemic symptoms (41). Studies suggest that EP may lead to serious complications such as heart failure and sepsis, which can be life-threatening in severe cases (4)Compared to other subtypes of psoriasis, patients with EP have a higher risk of mortality, making early disease control and the choice of effective treatment crucial. This study shows that IL-17-targeted biologics are highly effective in EP. A PASI 75 response rate of 78% (95% CI: 69%–86%) was achieved within 12 weeks of treatment, which was significantly higher than that of TNF-α and IL-23-targeted biologics. These results highlight the important role of IL-17-targeted biologics in the management of EP and support their use as a first-line treatment option for rapid and effective disease control.

Additionally, this study systematically evaluated treatment safety and found an overall adverse event incidence of 42% (95% CI: 21%–67%). Subgroup analysis by drug class showed distinct safety profiles: TNF-α inhibitors had the highest overall adverse event rate at 52% (95% CI: 4%–101%), followed by IL-17 inhibitors at 46% (95% CI: 18%–74%), while IL-23 inhibitors demonstrated the lowest rate of 5% (95% CI: –4%–13%). Although based on limited data, this finding is consistent with the favorable tolerability of IL-23 inhibitors observed in plaque psoriasis (45). The high target specificity of IL-23 inhibitors may contribute to this safety advantage by minimizing broad effects on immune function. For serious adverse events, incidence was 13% (95% CI: 4%–23%) with TNF-α inhibitors, 11% (95% CI: –1%–22%) with IL-23 inhibitors and 9% (95% CI: 0%–18%) with IL-17 inhibitors.

Despite differences in overall adverse event rates, the risk of serious adverse events remained relatively comparable across biologic classes and within an acceptably low range. These results suggest that biologics have an acceptable safety profile in EP treatment, with TNF-α-targeted agents exhibiting the most favorable safety. This information supports the selection of individualized treatment strategies in clinical practice.

This study has several limitations. First, the number of included studies was relatively small (n = 18), and most were observational rather than randomized controlled trials. This may reduce the precision of the pooled estimates and affect the reliability of subgroup analyses. Additionally, due to incomplete information on patients’ prior treatment history in the original study, we were unable to conduct a systematic stratified analysis between treatment-naive and treatment-experienced patients receiving biologics. This limitation may also influence the interpretation of efficacy and safety outcomes. Second, there were inconsistencies across studies in how efficacy endpoints such as PASI 75 and adverse events were defined, assessed, and reported. In particular, the documentation of mild to moderate adverse events varied considerably, which may have introduced heterogeneity, especially in the overall adverse event rate. In addition, follow-up duration differed among the included studies, limiting the assessment of long-term outcomes such as efficacy maintenance beyond 24 weeks, drug resistance, and delayed safety risks. Finally, some findings—such as the fluctuating efficacy of anti–TNF-α agents—differ from those typically reported in plaque psoriasis trials. This does not diminish the validity of our results but rather highlights that erythrodermic psoriasis, as a distinct and severe inflammatory condition, may lead to different biologic response patterns, influenced by factors such as high inflammatory burden, patient selection bias, and concomitant medications.

## 5 Conclusions

This meta-analysis indicates that biologics have favorable efficacy and safety in the treatment of erythrodermic psoriasis (EP), supporting their use in clinical practice. Comprehensive analysis shows that IL-17-targeted biologics produce a faster and stronger clinical response compared to other biologic types, while IL-23-targeted biologics demonstrate better safety. Based on current evidence, future large-scale studies with long-term follow-up are needed to further evaluate the efficacy and safety of different biologics in specific patient groups and to assess their role in long-term relapse prevention. Such research will help develop more individualized treatment strategies.

## Data Availability

The original contributions presented in the study are included in the article/**Supplementary Material**, further inquiries can be directed to the corresponding author/s.
