# Correction: Efficacy and safety of biologics in erythrodermic psoriasis: a systematic review and single-arm meta-analysis

**DOI:** 10.3389/fimmu.2026.1848885

**Published:** 2026-05-08

**Authors:** Lingjie Gao, Lu Shen, Hongwei Yan, Xinyang Liu, Yiran Wang, Xiaobo Li

**Affiliations:** 1 Department of Dermatology, The First Affiliated Hospital of China Medical University, Shenyang, China; 2 School of Nursing, China Medical University, Shenyang, China; 3 Shengjing Hospital Affiliated to China Medical University, Shenyang, China; 4 Nursing Department, The First Affiliated Hospital of China Medical University, Shenyang, China

**Keywords:** erythrodermic psoriasis, biologics, efficacy, safety, PASI 75

The article type “Review” was incorrectly chosen for this paper. The correct article type is “Systematic Review.”

As a result of the article type change, a **Data availability statement** has been inserted to read:

“The original contributions presented in the study are included in the article/supplementary material, further inquiries can be directed to the corresponding author/s.”

Affiliation 1 “Department of Dermatology, The First Affiliated Hospital of China Medical University, Shenyang, China” was erroneously given as “School of Nursing, China Medical University, Shenyang, China” and affiliation 2 “School of Nursing, China Medical University, Shenyang, China” was erroneously given as “Department of Dermatology, The First Affiliated Hospital of China Medical University, Shenyang, China”.

The original version of this article has been updated.

