# Supplementary material for: Efficacy and safety of biologics in erythrodermic psoriasis: a systematic review and single-arm meta-analysis
Source: Front Immunol. 2025 Oct 29;16:1714587. doi: 10.3389/fimmu.2025.1714587 (PMC12605349; doi:10.3389/fimmu.2025.1714587)
Supplement: Supplementary file 1 [file DataSheet1.docx]

Supplementary Material

# Supplementary Data

# Supplementary Figures and Tables

## Supplementary Figures


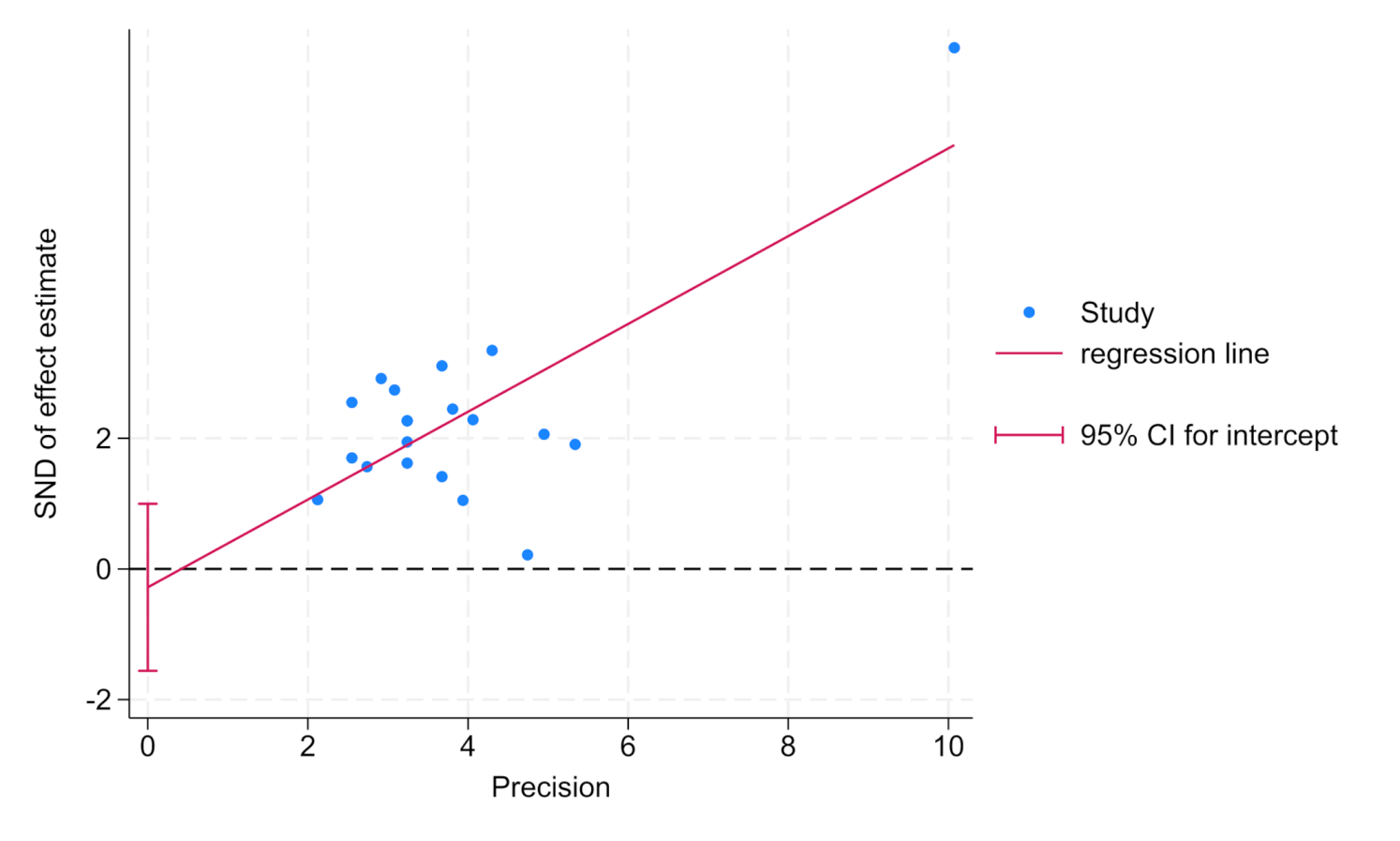
**Supplementary Figure 1.** Egger Test Plot for PASI 75 Response Rate Within 12 Weeks.


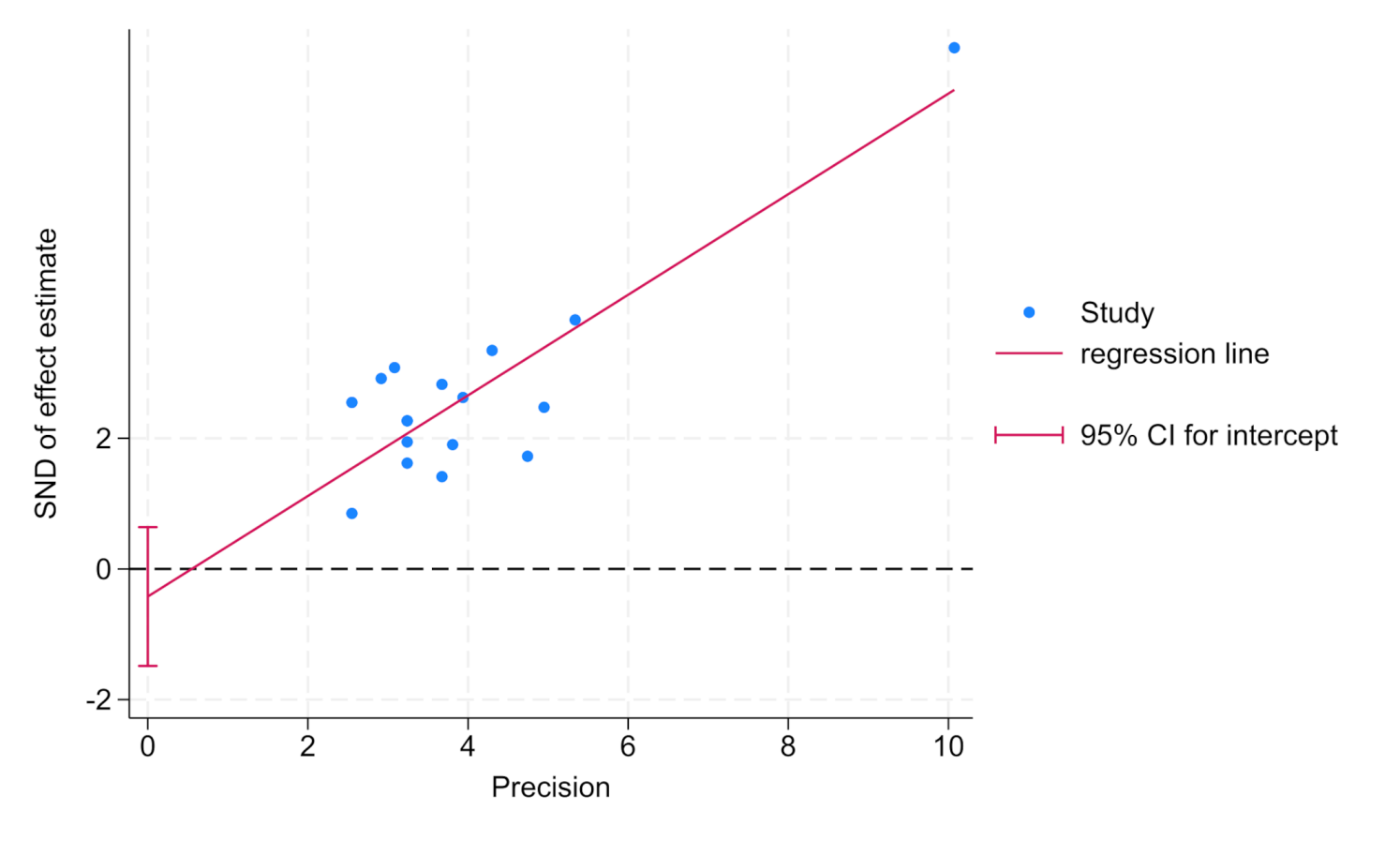


**Supplementary Figure 2.** Egger Test Plot for PASI 75 Response Rate Within 16 Weeks.


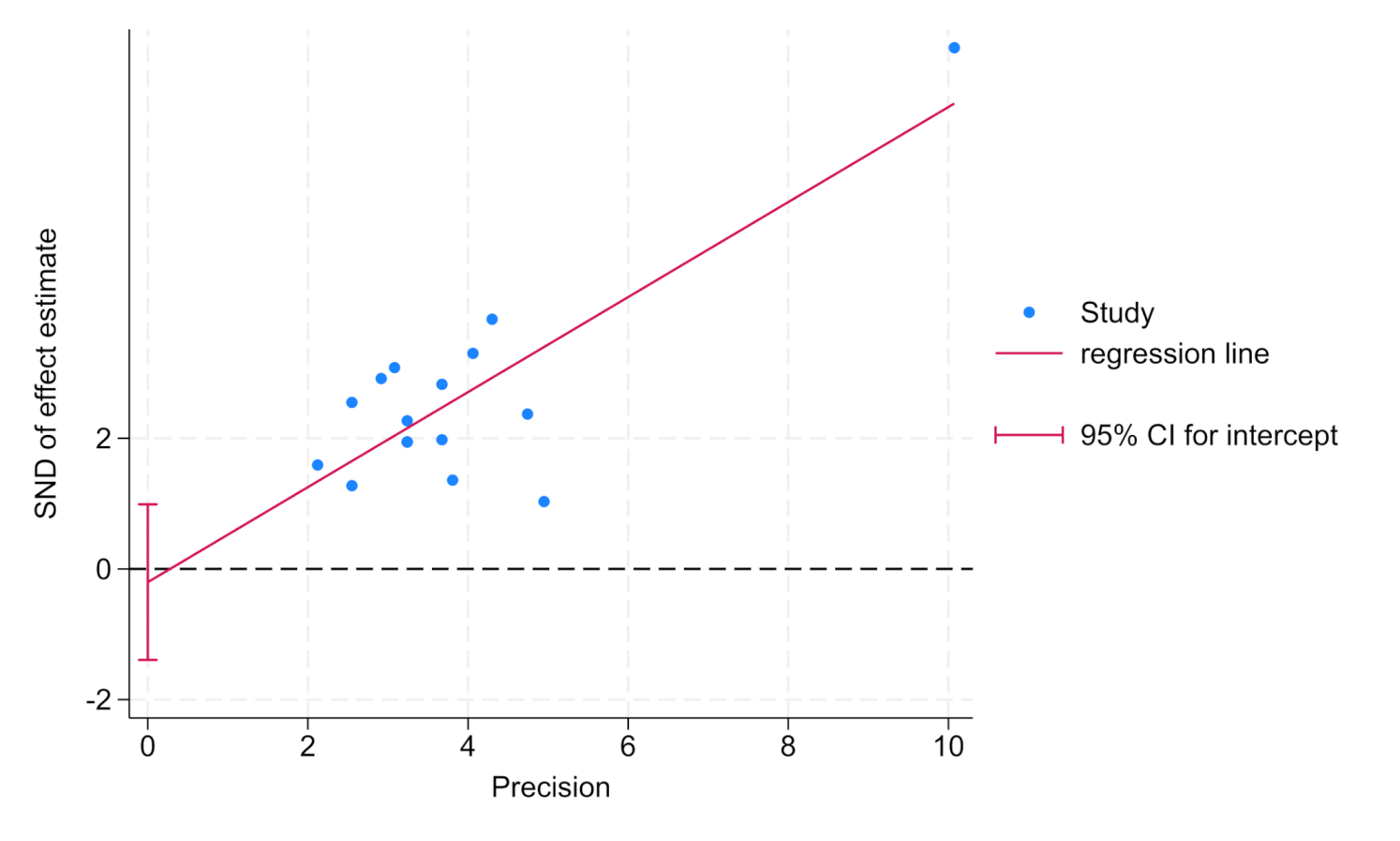


**Supplementary Figure 3.** Egger Test Plot for PASI 75 Response Rate Within 24 Weeks.


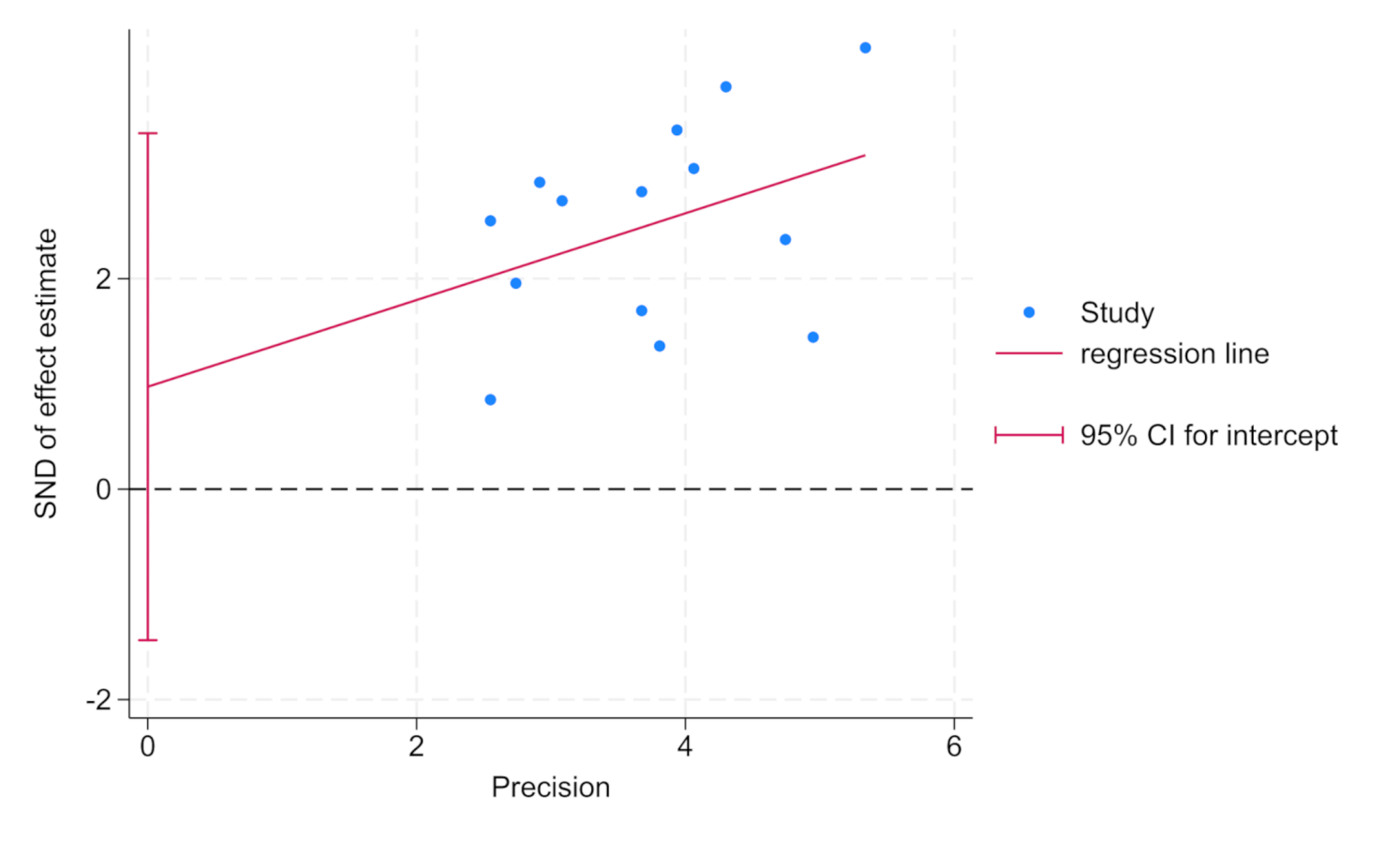


**Supplementary Figure 4.** Egger Test Plot for PASI 75 Response Rate after 24 Weeks.


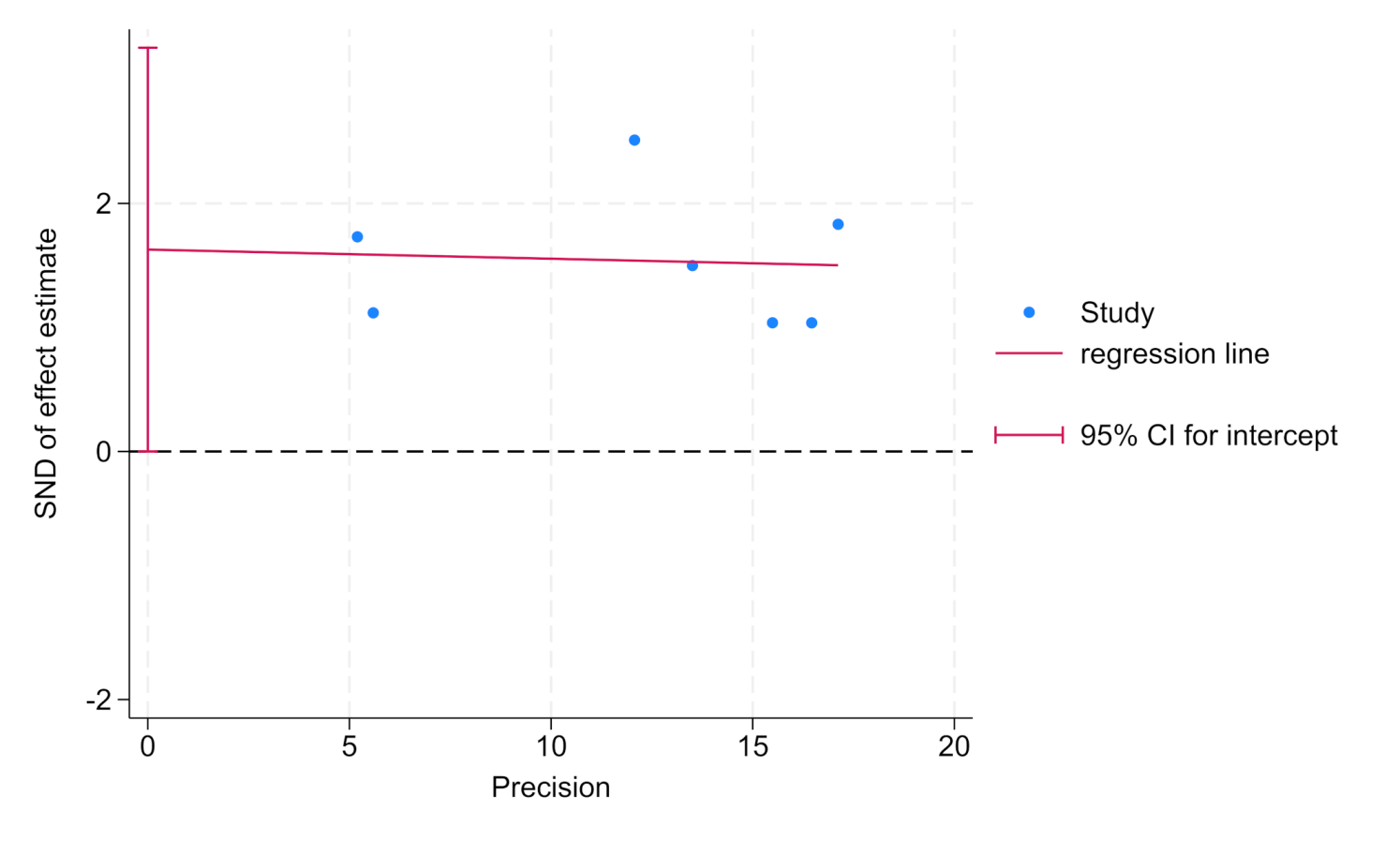


**Supplementary Figure 5.** Egger Test Plot for Serious Adverse Events


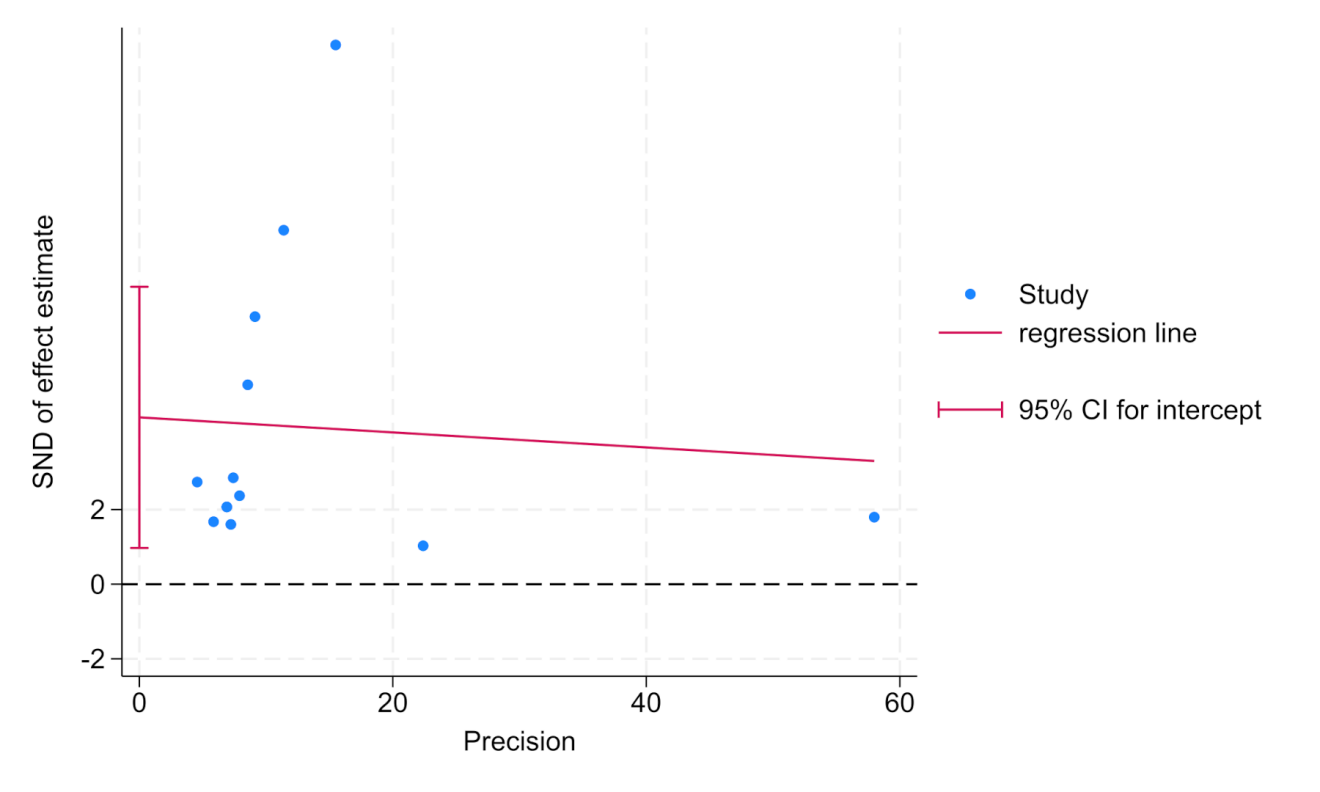


**Supplementary Figure 6.** Egger Test Plot for Adverse Events

## Supplementary Tables

**Table 1. Pubmed Search strategy**

| Item | Search strategy | n |
| --- | --- | --- |
| #1 | "Psoriasis"[MeSH Terms] | 51988 |
| #2 | "erythroderm*"[Title/Abstract] | 4262 |
| #3 | #1 AND #2 | 728 |
| #4 | "erythrodermic psoriasis"[Title/Abstract] OR "exfoliative psoriasis"[Title/Abstract] OR "generalized erythroderma"[Title/Abstract] | 540 |
| #5 | #3 OR #4 | 971 |
| #6 | "biologic*"[Title/Abstract] OR "Etanercept"[Title/Abstract] OR "Infliximab"[Title/Abstract] OR "Adalimumab"[Title/Abstract] OR "golimumab"[Title/Abstract] OR "certolizumab"[Title/Abstract] OR "Secukinumab"[Title/Abstract] OR "Ixekizumab"[Title/Abstract] OR "brodalumab"[Title/Abstract] OR "Guselkumab"[Title/Abstract] OR "Ustekinumab"[Title/Abstract] OR "risankizumab"[Title/Abstract] OR "Tildrakizumab"[Title/Abstract] OR "spesolimab"[Title/Abstract] OR "gevokizumab"[Title/Abstract] OR "canakinumab"[Title/Abstract] OR "Bimekizumab"[Title/Abstract] OR "Tildrakizumab"[Title/Abstract] OR "tnf alpha inhibitors"[Title/Abstract] OR "il 17 inhibitors"[Title/Abstract] OR "il 23 inhibitors"[Title/Abstract] OR "il 36 receptor antagonists"[Title/Abstract] OR "il 12 inhibitors"[Title/Abstract] | 1249637 |
| #7 | #5 AND #6 | 216 |
